# Pulmonary melioidosis in CAMBODIA: a prospective study

**Published:** 2011-01-10

**Authors:** Blandine Rammaert, Julien Beauté, Laurence Borand, Sopheak Hem, Philippe Buchy, Sophie Goyet, Rob Overtoom, Cécile Angebault, Vantha Te, Patrich Lorn Try, Charles Mayaud, Sirenda Vong, Bertrand Guillard

**Affiliations:** 1 Epidemiology and Public Health Unit, Institut Pasteur in Cambodia, Phnom Penh, Cambodia; 2 Biomedical Laboratory, Institut Pasteur in Cambodia, Phnom Penh, Cambodia; 3 Virology Unit, Institut Pasteur in Cambodia, Phnom Penh, Cambodia; 4 Swiss Red Cross, Takeo, Cambodia; 5 Pediatric Department, Donkeo Provincial Hospital, Takeo, Cambodia; 6 Pediatric Department, Provincial Hospital, Kampong Cham, Cambodia; 7 Pneumology and Intensive Care Unit, Hôpital Tenon, Paris, France

## 

Melioidosis is a disease caused by the soil-dwelling Gram-negative bacterium *Burkholderia pseudomallei*. It is endemic in South-East Asia but remains poorly documented in Cambodia where laboratory facilities are scarce. We report here a cohort of culture-confirmed cases of pulmonary melioidosis identified in two provincial hospitals in Cambodia, describing clinical and epidemiological characteristics.

Patients with melioidosis were identified through a laboratory based surveillance of acute lower respiratory infections (<14 days of illness) in two provincial hospitals from April 2007 to January 2010. *B. pseudomallei* was detected in sputum or blood through 42 cultures and confirmed by API 20 NE gallery. We collected clinical, microbiological and radiological data and visited patients several weeks after hospital discharge to document long-term outcome.

Melioidosis was found in 39 patients. The median age was 46 years including three patients ≤2 years and 56.4% were males. A close contact with soil and water was identified in 30 patients (76.9%). Pneumonia was the main radiological feature (82.3%), but pleurisy was also described in 6 patients. Eleven patients were severe. A positive blood culture was significantly associated with severe cases (90.9% vs. 50.0%; p<0.05) and with higher fatality (87.5% vs. 20%; p<0.01). A total of 24 (61.5%) patients died within 3 days, 23 without receiving any active drug against *B. pseudomallei*. One year after discharge, 11 patients were still alive and considered as cured.

Melioidosis is an emerging public health issue in Cambodia that requires nationwide access to laboratory facilities and timely appropriate treatment.

